# An Updated Update to Personality and Error Monitoring

**DOI:** 10.3389/fnhum.2012.00283

**Published:** 2012-10-17

**Authors:** Mattie Tops, Sander L. Koole

**Affiliations:** ^1^Department of Clinical Psychology, VU University AmsterdamAmsterdam, Netherlands; ^2^Centre for Child and Family Studies, University of LeidenLeiden, Netherlands; ^3^Leiden Institute for Brain and Cognition, Leiden University Medical CenterLeiden, Netherlands

People differ strongly in the degree of error processing, and how errors are interpreted and appraised. In a recent study in Frontiers in Human Neuroscience, Hoffmann et al. (2012) investigated whether a correlate of error monitoring, the error negativity (Ne or ERN), is related to personality factors. They measured the EEG continuously during a task that provoked errors, and the Ne was tested with respect to its relation to personality traits. The amplitude of the Ne was smaller in individuals who scored higher on the “Openness” scale, the “Impulsiveness” scale, and the “Emotionality” scale. By contrast, the Ne was larger in individuals who scored higher on the “Social Orientation” scale. These results are partly consistent with previous studies of associations between Ne and personality, and extent those associations to traits that had not been investigated before in this context. However, Hoffmann and colleagues missed some recent findings that may be important in the interpretation of their results.

Previous studies associated Ne amplitude with various traits that often seemed very different from each other. We recently convincingly demonstrated that one thing those traits have in common is that they predict task engagement, suggesting that task engagement is a common underlying factor that predicts the amplitude of the Ne (Tops and Boksem, 2010). In a two-study paper, we first showed that the traits that have been related to Ne amplitude in previous research are interrelated and have in common that they are correlated with the motivational trait of *Persistence*. This by itself supports the hypothesis that engagement is a common underlying factor that predicts the amplitude of the Ne. An alternative factor, such as *concern over social evaluation*, may relate to Persistence and may perhaps explain the association of traits such as BIS and neuroticism with persistence, but does not seem involved in obvious ways in some of the other traits, such as Drive for reward, Impulsivity, and Absorption. Moreover, the second study provided additional support for the engagement hypothesis by showing that the traits interact with context to predict the Ne, such that trait–context combinations that are likely to be associated with increased engagement predict larger Ne amplitudes. For instance, a trait measure of intrinsic motivation (Absorption) predicted both larger Ne amplitudes during the first part of performance when boredom had not yet set in, and a larger decrease in amplitudes during later performance. By contrast, Constraint, a trait related to the resistance of temptation and distraction, predicted larger Ne amplitudes only during later performance when boredom and fatigue increased temptations to disengage. We also review evidence that externalizing psychopathological syndromes in which reduced Ne amplitudes have been found are characterized by reduced Persistence, while internalizing syndromes such as obsessive compulsive disorder in which increased Ne amplitudes have been found are characterized by increased Persistence (Tops and Boksem, 2010).

The traits investigated by Hoffmann and colleagues are related to previously investigated traits. For instance, “Impulsiveness” is associated with externalizing and the opposite pole of Constraint. “Openness” (vs. following social norms and making good impression) and “Social orientation” (helpful vs. uncooperative) are both very likely correlates of the Agreeableness trait that we showed to relate positively to task engagement, trait Persistence, and Ne amplitude (Tops et al., 2006; Tops and Boksem, 2010). Indeed, in their Discussion, Hoffmann and colleagues argued themselves that their traits were interrelated and may be associated with the level of engagement during task performance. Although this suggests that task engagement may provide a parsimonious account for individual differences in Ne amplitudes at the trait and state levels, it cannot be ruled out that mechanisms behind the Ne are functionally implicated in negative affect or behavioral inhibition (e.g., Tops and Boksem, 2011). More studies are needed to address this unresolved issue.

One part of the results of Hoffmann and colleagues that appears to deviate from previous findings is their finding of an association between “Emotionality” and smaller Ne amplitudes. Many studies related Ne amplitude to individual differences that reflect anxiety, punishment sensitivity, or negative emotion traits and it has been suggested that the Ne reflects concern with the outcome of events, which may increase engagement (Hajcak et al., 2005; Boksem et al., 2006, 2008; Tops et al., 2006; Santesso and Segalowitz, 2009). However, it was recently found that Ne amplitude does not relate to such individual differences if trial-by-trial performance feedback is provided (Olvet and Hajcak, 2009). This could mean that also in the Hoffmann et al. study, the provision of performance feedback may have altered performance monitoring processes and error-related potentials because it was possible to rely on the performance feedback to monitor performance accuracy. However, significant Ne effects showed that there was error processing before feedback. Possibly, in the local-global task, some baseline level of performance monitoring is performed regardless of the presence of feedback, and is sufficient to detect error responses; the feedback may decrease excessive error-monitoring or error-related orienting responses that are related to anxious and emotional traits, or may increase anticipatory processes related to potentially distressing feedback.

To conclude, future studies of individual trait and state differences in Ne amplitude should measure and/or manipulate task engagement to help interpret results and to investigate potential additional determinants of Ne amplitude besides engagement. Among the factors that can be used for this purpose are measurements of traits such as Persistence, variations in task demands, task length and motivation, and interaction between relevant traits and conditions (Tops and Boksem, 2010).
